# Consultations for refractory cases in mental health services: a descriptive study

**DOI:** 10.1186/s12888-023-04559-5

**Published:** 2023-01-27

**Authors:** B. Stringer, R. J. T. Mocking, D. Rammers, B. Koekkoek

**Affiliations:** 1 Center for Consultation and Expertise, Utrecht, The Netherlands; 2 Department of Psychiatry, Amsterdam University Medical Centers, Location AMC, University of Amsterdam, Amsterdam, The Netherlands; 3 Research Group for Social Psychiatry and Mental Health Nursing, HAN University of Applied Science, Nijmegen, The Netherlands; 4 Pro Persona Mental Health Services, Wolfheze, The Netherlands; 5 Research Department, Police Academy, Apeldoorn, The Netherlands

**Keywords:** Refractory cases, Consultation, Challenging behavior, Mental health services

## Abstract

**Background:**

Yearly, almost six percent, which is more than 1,000.000 people, in the Netherlands receive mental health treatment, which usually improves their quality of life. Concurrently, mental healthcare professionals recognize clinically refractory cases in which improvement fails to occur, with severe ongoing burdens for patients. The Dutch Centre for Consultation and Expertise (CCE) is available to support such refractory cases. The Dutch government’s (financial) facilitation of consultation through the CCE is unique in the world. CCE consultations provide therefore unique insight into and an overview of refractory cases in mental health services. The objective of this study was to gain insight into the commonalities underlying the reasons for CCE consultations and the solutions proposed that play roles in (the reduction of) refractory cases for which consultation has been requested.

**Methods:**

This descriptive study was conducted with quantitative and qualitative data from 472 CCE consultations in the Netherlands. Using descriptive statistics and thematic content analysis, four exemplary situations were distilled from the qualitative data.

**Results:**

Most (83%) cases in the sample could be explained with four exemplary situations involving self-harm (24.2%), aggression (21.8%), self-neglect (24.4%), and socially unacceptable behavior (12.5%), respectively. Each situation could be characterized by a specific interaction pattern that unintentionally maintained or aggravated the situation. At the time of closure of the consultation applicants’ questions had been answered and their situations had improved in 60.4% of cases.

**Conclusions:**

This study offers an overview of approaches that provided new perspectives for patients and professionals in many refractory cases in the Dutch mental health services.

## Background

Mental health services (MHS) usually contribute to patients’ recovery and quality of life improvement [1]. However, practice guidelines do not provide solutions when patients’ improvement is insufficient [2–4]. Several authors have described preconditions and models for (mental health) consultation in such situations [5, 6], but research on the results of such consultation is limited and mental healthcare consultation content has not been evaluated systematically.

The Dutch government established a Centre for Consultation and Expertise (CCE) in 1989 to provide support for exceptionally challenging clinical situations involving patients dependent on long-term professional care. The CCE is positioned uniquely in the healthcare system; it provides free-of-charge services supplementary to standard health services and is funded directly by the Ministry of Health. Individuals requiring long-term care whose quality of life is at risk of being seriously compromised, or their caregivers or next of kin, can apply to the CCE for “exceptional care”. The CCE works with independent experts and provides customized advice and support. The CCE's view is that challenging behavior is not a patient factor per se, but should always be seen in terms of interaction with the patients’ physical and social contexts [3, 7]. The main focus is on understanding the refractory situation from multiple perspectives and searching for dysfunctional patterns in the patient–​​healthcare team–next-of-kin triad. CCE experts can advise on additional or missing diagnostics, but do not conduct these examinations themselves.

The CCE was initially focused on the intellectual disability sector, but since 2008 its scope has broadened to include MHS and services for troubled youth and older adults. The CCE is consulted about 1500 times annually, including about 300 MHS consultations.

To our knowledge, this national center providing consultation for refractory cases in the MHS is unique in the world. Thus, an analysis of CCE consultations may provide valuable insight into the situations in which MHS are unable to provide adequate care and consultation is sought. Moreover, the CCE initiative in the Netherlands may represent a way to adequately address these situations in healthcare services, and thus may provide inspiration for other national healthcare systems. In this retrospective file study, we examined mental health–related CCE consultations to address the following research questions:

- What situations lead to CCE consultation, reflecting the inability of standard MHS to provide adequate treatment?
- What patient and context variables underlie these situations?
- What possible solutions does the CCE propose in these situations?

The overall aim was to gain insight into the commonalities underlying the reasons for mental health-related CCE consultations and the solutions proposed that play roles in (the reduction of) refractory cases for which consultation has been requested.

## Methods

### Sample

The CCE uses a client tracking system (CTS) in which files on all consultations are kept. We included mental healthcare–related CCE consultations requested for patients aged 18–65 years between 1 January 2016 and 1 July 2019 in this study. We excluded requests for which no consultation was initiated after initial assessment due to relocation, patient death, non-consent, lack of applicant response despite repeated contact or improper request. Finally, we included 472 files for this study.

### Data collection

For this retrospective study, we used quantitative variables generated automatically and anonymously from the CTS, including demographic, process, context, and patient variables (see Table 1). In addition, the CTS contains qualitative summaries written at the time of file closure, each with a description of 1) the reason for consultation, 2) CCE experts’ explanation of the problem, 3) the proposed solutions, and 4) results. The responsible coordinator of each case file wrote these summaries. We extracted the verbatim data from these open text fields.

**Table 1:** Overview of patient characteristics^a^

Characteristic	Total	Self-harm	Aggression	Self-neglect	Socially unacceptable behavior	Other
*n* (%)	472 (100)	114 (24.2)	103 (21.8)	115 (24.4)	59 (12.5)	81 (17.2)
Sex [*n* (%)]						
Male	262 (55.5)	18 (15.8)	78 (75.7)	92 (80)	32 (54.2)	42 (51.9)
Female	207 (43.9)	95 (83.3)	24 (23.3)	22 (19.1)	27 (45.8)	39 (48.1)
*Missing*	*3 (0.6)*					
Age [years; mean ± SD (range)]	35.8 ± 12.93 (19–64)	30.7 ± 11.1 (19–58)	37.1 ± 12.4 (19–64)	38.8 ± 12.7 (19–64)	37.9 ± 13.4 (19–64)	35.4 ± 13.4 (19–63)
Duration of consultation process (months; mean ± SD)	10.2 ± 6.6	10.0 ± 6.7	9.4 ± 6.4	10.9 ± 6.7	11.2 ± 7.2	9.1 ± 5.6
Applicant [*n* (%)]						
Patient	36 (7.6)	16 (14.0)	0 (0.0)	9 (7.8)	1 (1.7)	10 (12.3)
Patient representative	62 (13.1)	14 (12.3)	6 (5.8)	24 (20.9)	1 (1.7)	17 (21.0)
Healthcare provider	338 (71.6)	76 (66.7)	93 (90.3)	77 (67.0)	51 (86.4)	41 (50.6)
Other	42 (7.9)	6 (5.3)	4 (3.9)	5 (4.3)	5 (8.5)	12 (14.8)
*Missing*	*4 (0.8)*					
Reason for request [*n* (%)]						
Inability to provide adequate care	323 (68.4)	87 (76.3)	76 (73.8)	85 (73.9)	39 (66.1)	36 (44.4)
Lack of proper care provision	120 (25.4)	18 (15.8)	23 (22.3)	26 (22.6)	19 (32.2)	34 (42.0)
Stagnating dialogue	22 (4.7)	5 (4.4)	3 (2.9)	4 (3.5)	1 (1.7)	9 (11.1)
*Missing*	*7 (1.5)*					
Reason for closure [n (%)]						
Issue resolved	285 (60.4)	60 (52.6)	58 (56.3)	85 (73.9)	41 (69.5)	41 (50.6)
Discontinued by healthcare provider	51 (10.8)	14 (12.3)	13 (12.6)	6 (5.2)	5 (8.5)	13 (16.0)
Discontinued by CCE	31 (6.6)	7 (6.1)	6 (5.8)	5 (4.3)	2 (3.4)	11 (13.6)
Relocation	48 (10.2)	10 (8.8)	15 (14.6)	11 (9.6)	6 (10.2)	6 (7.4)
Patient death	12 (2.5)	9 (7.9)	0 (0.0)	1 (0.9)	1 (1,7)	1 (1,2)
Waiting for follow-up	45 (9.5)	14 (12.3)	11 (10.7)	7 (6.1)	4 (6.8)	9 (11.1)
Legal status [n (%)]						
Voluntary	153 (32.4)	46 (40.4)	18 (17.5)	42 (36.5)	15 (25.4)	32 (39.5)
Taken into custody^b^	18 (3.8)	6 (5.3)	6 (5.8)	3 (2.6)	2 (3.4)	1 (1.2)
Judicial authorization^b^	121 (25.6)	26 (22.8)	37 (35.9)	30 (26.1)	21 (35.6)	7 (8.6)
Placed under legal restraint^c^	2 (0.4)	0 (0.0)	2 (1.9)	0 (0.0)	0 (0.0)	0 (0.0)
Other	35 (7.4)	5 (4.3)	11 (10.6)	9 (7.8)	6 (10.2)	4 (4.9)
*Missing*	*143 (30.3)*					
Restrictive measures [n (%)]	79 (16.7)	16 (14.0)	41 (39.8)	8 (7.0)	5 (8.5)	9 (11.1)
Setting [n (%)]						
Inpatient	149 (31.6)	37 (32.4)	53 (51.5)	32 (27.8)	17 (28.8)	10 (12.3)
Forensic inpatient	41 (8.7)	7 (6.1)	20 (19.4)	7 (6.1)	6 (10.2)	1 (1.2)
Outpatient	179 (38.0)	41 (35.9)	15 (14.6)	61 (53.0)	24 (40.7)	38 (46.9)
Other	33 (7.0)	11 (9.6)	6 (5.8)	7 (6.1)	3 (5.1)	6 (7.4)
Unknown	70 (14.8)					
Cognitive level [n (%)]						
Normal (> 85)	235 (49.8)	72 (63.2)	46 (44.7)	71 (61.7)	11 (18.6)	35 (43.2)
Borderline intellectual functioning (70–84)	69 (14.6)	10 (8.8)	16 (15.5)	11 (9.6)	22 (37.2)	10 (12.3)
Mild intellectual disability (50–69)	40 (8.5)	6 (5.3)	12 (11.7)	8 (7.0)	11 (18.6)	3 (3.7)
Severe intellectual disability (< 34–49)	8 (1.7)	0 (0.0)	2 (1.9)	2 (1.8)	1 (1.7)	3 (3.7)
*Missing*	*140 (29.7)*					
Problem behavior [n (%)]						
Aggressive behavior	160 (33.9)	21 (18.4)	69 (67.0)	37 (32.2)	23 (39.0)	10 (12.3)
Problematic verbal behavior	101 (21.4)	15 (13.2)	38 (36.9)	27 (23.5)	11 (18.6)	11 (18.6)
Self-harm	75 (15.9)	52 (46.5)	6 (5.8)	5 (4.3)	5 (8.5)	6 (7.4)
Sexually transgressive behavior	38 (8.0)	5 (4.4)	14 (3.6)	4 (3.5)	10 (16.9)	5 (6.2)
Oppositional behavior	86 (18.2)	17 (14.9)	30 (29.1)	18 (15.7)	12 (20.3)	9 (11.1)
Extremely passive behavior	69 (14.6)	13 (11.4)	7 (6.8)	32 (27.8)	7 (11.9)	10 (12.3)
Extremely active behavior	10 (2.1)	0 (0.0)	4 (3.9)	1 (0.9)	4 (6.8)	1 (1.2)
Destructive behavior	84 (17.8)	19 (16.7)	28 (27.2)	14 (12.2)	14 (23.7)	9 (11.1)
Suicidal behavior	49 (10.4)	15 (13.2)	13 (12.3)	9 (7.8)	8 (13.6)	4 (4.9)
Other	58 (12.3)	11 (9.6)	5 (4.9)	20 (17.4)	12 (20.3)	10 (12.3)
Psychiatric problems at start of consultation [n (%)]						
Addiction	81 (17.2)	9 (7.9)	25 (24.3)	24 (20.9)	19 (32.2)	4 (4.9)
Psychotic spectrum disorder	165 (35.0)	9 (7.9)	64 (62.1)	53 (46.1)	23 (39.0)	16 (19.6)
Personality disorder	119 (25.2)	55 (48.2)	18 (17.5)	14 (12.2)	18 (30.5)	14 (17.3)
Autism Spectrum Disorder	167 (35.4)	35 (30.7)	36 (35.0)	58 (50.4)	10 (16.9)	28 (34.6)
Mood disorder	93 (19.7)	43 (37.7)	9 (8.7)	19 (16.5)	12 (20.3)	10 (12.3)
Attachment problems	35 (7.4)	15 (13.2)	6 (5,8)	4 (3,5)	5 (8.5)	5 (6.2)
Eating disorder	46 (9.7)	32 (28.1)	1 (1.0)	5 (4,3)	3 (5.1)	5 (6.2)
Anxiety disorder	60 (12.7)	21 (18.4)	4 (3,9)	17 (14.8)	6 (10.2)	12 (14.8)
ADHD^d^	22 (4.7)	6 (5.3)	5 (4.9)	5 (4.3)	2 (3.4)	4 (4.9)
Other	19 (4.0)					

^a^Data are derived from the CCE’s client tracking system

^b^Under the Dutch Mental Health Act

^c^Under the Dutch Forensic Health Act

^d^Attention Deficit Hyperactivity Disorder

### Data analysis

We calculated descriptive statistics for the quantitative data on situations for which consultation was requested. Using thematic content analysis [8], we distilled exemplary situations from the qualitative data. In an exploratory analysis (BS), open coding was applied to the data to identify categories in’reasons for consultation’. Subsequently, the analysis focused to the identification of recurring themes in the CCE experts’ problem explanations and proposed solutions, as well as common threads in the described consultation results (BS). In this process the original categories were merged, based on the analysis of the problem explanations. If the ‘request for consultation’ categories overlapped, the problem explanation determined the definitive category. The coding and categories were reviewed with the co-authors (RM, BK, and DR). Two groups of five internal and six external experts then reviewed the categories in more detail, leading to revision of the descriptions (by BS and DR). In a final peer debriefing step with co-authors (BS, RM, and BK), we definitively defined the exemplary situations. We used triangulation of the quantitative and qualitative findings to further validate the exemplary situations.

## Results

### Quantitative findings

The original sample comprised 535 files; application of the exclusion criteria led to the exclusion of 63 consultations. Reasons for exclusion were 20% relocation, 5% deaths, 8% non-consent, 44% lack of response and 22% improper request, respectively. Thus, 472 consultations concerning 462 individuals were included. Reasons for consultations were: general inability to provide adequate care (68,4%), lack of appropriate healthcare provision (25.4%) and conflict between the parties involved (4.7%). Applicants’ questions had been answered and the situations had improved at the time of closure in 60.4% of cases (Table 1).

### Qualitative findings

Initially, 16 categories of refractory situation were identified. Ultimately, the analytical process yielded four exemplary situations of refractoriness that typified the inability to provide adequate care, leading to consultation requests. Together, these situations accounted for 82.8% of all consultations. The remaining 17.2% of situations, covered by the residual category, involved specific symptomatologies (e.g., conversion disorder, medically unexplained physical symptoms, Korsakov syndrome, and early dementia symptoms). The four exemplary situations centered on self-harm, aggression, self-neglect, and socially unacceptable behavior.

#### Self-harm

In this situation patients damaged themselves, and treatment teams lost their positivity toward them. Most patients in this situation were young women with average cognitive function with extreme and prolonged (self-)destructive behavior included self-harm, suicidal behavior, and/or severe underweight. According to their treatment teams, these patients had high estimated risk of death, and therapies had yielded no or insufficient results. The teams and family members had become demoralized, exhausted and felt powerless by the situation. Thus, they often could not approach the patients in a neutral or positive way, which aggravated the patients’ problems. At the start of consultation, the treatment team explained the patients’ challenging behavior from the perspective of a personality or mood disorder. Many professionals focused on patients’ autonomy and policies for high-risk patients (in the outpatient context) or on security, with increasing implementation of repressive measures in the effort to make the patients’ self-harming behavior manageable (in the inpatient context).

Quote: “Normally gifted woman who is accompanied from the seclusion room. She shows a lot of destructive, aggressive and self-harming behavior. The team feels powerless and they are unable to get her into a more normalized living environment.”

Problem explanations suggested by the CCE experts focused on existing patient–team interactions. Team members’ increasing despair and feelings of powerlessness about the patients’ serious self-harm caused them to lose sight of the broader context of the patients’ challenging behavior.

CCE experts pointed out that the emphasis on patients’ autonomy led to overestimation of the patients, resulting in increased stress. They noted that self-harm was the main way in which patients handled stress, which led to healthcare professionals’ despair, frustration, and inability to remain neutral or positive when interacting with these patients, who in turn experienced even more stress and persisted with coping through self-harm. According to the CCE experts, healthcare professionals appealed to the patients’ autonomy, seeking to make them responsible for their safety, but the patients could not bear such responsibility. At the patient level, previously unrecognized autism spectrum disorder (ASD) was often diagnosed during the consultations.

Quote: “People have always assumed a borderline personality disorder, but it turns out to be ASD and a very low social-emotional level with attachment problems. Previously, the responsibility for her behavior was placed with the patient. Now, the behavior is being seen as appropriate for her low social-emotional functioning. The interpersonal way of approaching her has been adapted accordingly.”

In terms of solutions, the CCE experts paid ample attention to the teams’ negative emotions and experience of powerlessness. These experts suggested that the overestimation of patients as a result of the overemphasis on autonomy could be reduced by introducing into the situation a “trusted other” – a familiar, stable, and predictable personal care attendant – for each patient.

*Quote: “*New* team deployed to guide her and then transfer to more normalized home. One-on-one guidance. New problem explanation was shared with new team.”*

These trusted others would offer closeness and undertake successful activities together with the patients, appealing to the patients’ psychological strengths. CCE experts pointed out the importance of the unconditionality of these relationships, i.e., the need to focus continuously on the reintegration of normal rhythms and activities in a normal environment, regardless of patients’ self-harm. Giving less (negative) attention to patients’ self-harm ultimately contributed to the reduction of their stress.

*Quote: “Less self-harm, less aggression and destruction, more day-to-day activities. Medication has been almost completely reduced. Quality of Life increased.”*

#### Aggression

The second refractory situation focused on aggression, in which involuntarily admitted patients harmed other people and teams increasingly put distance from patients, with physical isolation of the patients as a result. Most patients in this situation were men with long histories of involuntary MHS admissions and occasional transfers to forensic mental health facilities or prison. Frequent and serious aggression incidents often led to long-term individual supervision or seclusion. In some cases, patients’ aggression was provoked by substance abuse. Patients showed little or no response to medication. The professionals responsible for these patients usually described their aggression as unpredictable and the patients as inscrutable. Teams responsible for their care became anxious and exhausted. Patients’ family members often had been distanced from them during their long periods of illness.

*Quote: “Patient has a long history of psychosis, behavioral and serious addiction problems. The patient also has judicial contacts with regard to aggression, vandalism, threats, for which various punitive measures and placements within forensic psychiatric departments. Now admitted at High and Intensive Care unit and not following the program, walking a lot and suffers from craving.”*

At the start of consultation, professionals explained the patients’ challenging behavior from the perspective of psychotic or schizophrenic disorders or ASD, in combination with addiction problems and borderline intellectual functioning or mild intellectual disability. These multimorbidities served as explanations for the failure to control the patients’ strong aggression. Partly for this reason, highly secure environments were considered to be necessary to guarantee the security of other patients and team members.

Problem explanations suggested by CCE experts focused on existing team–patient interactions: a pattern of resistance regularly arose among team members. Due to patients’ aggression, team members became anxious and kept more physical and social distance from the patients. Contact became mainly functional, leaving the patients increasingly to the mercy of their overwhelming inner worlds, which resulted in stress. The patients used aggression to cope with this stress, increasing team members’ distancing. The CCE experts often observed that the patients had disharmonious developmental profiles, with a lag especially in social-emotional development (SED). This insufficient SED alignment caused overestimation of these patients, leading to stress and thus aggression. For patients with (unrecognized) ASD, psychotic transgressions often could be explained by (prolonged) overestimation. *Quote: “Hypothesis is overestimation due to low social-emotional level”.*

Acquired brain damage was also frequently diagnosed during consultation. Acquired brain damage has major consequences for daily functioning, but these consequences were not always sufficiently taken into account.

In terms of proposed solutions CCE experts pointed out that a more proactive interpersonal approach might break the patterns resulting from interaction with these patients; more closeness with trusted professionals could regulate the behavior and emotions of patients with low SED. In this closeness, the focus was on “subtitling” social interactions and performing joint activities – initially with individual supervision – so that patients gained positive experiences again. These patients were often far removed from ordinary life due to histories of long-term seclusion. Investment in the normalization of their environments and daily routines also reduced their stress and thus their aggression.

*Quote: “Clear what patient needs.”*

The medication reviews performed as part of the consultations regularly led to medication reduction for patients with ASD or unrecognized mild intellectual disability and acquired brain damage. Cases of undermedication were also identified when the professional–patient–family triad had not agreed on the medication strategy. The consultants’ input regularly ensured that these triad reached agreement on this issue.

#### Self-neglect

The third refractory situation focused on patients who neglected themselves, whom teams consequently lost contact with. Most patients in this situation were somewhat eccentric men who allowed minimal care provision. They were often evasive and repulsive during contact (i.e., expressing physical and verbal aggression), with behaviors that were difficult to interpret. They often exhibited compulsions or fear, which were poorly understood, leading to professionals’ and patients’ frustration. Patient–healthcare professional relationships were characterized by little reciprocity, generating the risk that healthcare professionals avoided or refused to take care of them, causing the patients to self-neglect further. Many of these patients regularly used illegal drugs as self-medication. The professionals often concluded that the patients “did not fit” well in e.g. the sheltered housing institutional context. The patients’ next of kin felt powerless because they sensed that their family members were not well understood. At the start of consultation, the professionals responsible for these patients explained their challenging behavior via ASD or psychotic disorders and anxiety. They felt powerless to influence these patients’ self-neglect.

*Quote: “Man living in a sheltered housing facility, who rejects all care from professionals. He can react aggressively if they approach him according to house rules. Seems to lose grip on reality, extremely anxious and threatening when approached.”*

Problem explanations suggested by CCE experts focused on the overemphasis on the demand for these patients’ autonomy, as they were working hard to merely survive. These patients’ rejection of contact was often explained by insufficient understanding of how people with ASD view the world. Many of these patients also had disharmonious developmental profiles. In terms of possible solutions psychoeducation about ASD and disharmonious developmental profiles for teams, patients, and their next of kin increased insight into these patients’ situations and led to a more proactive style of counseling; limiting the number of choices and dividing activities into more concrete and manageable segments with instructions gave patients more control. The search for and deployment of motives in the patients’ lives had been neglected due to prolonged impotence on the part of all parties. Becoming curious about these motives again often provided new openings for positive contact.

*Quote: “ASD, probably psychotic symptoms when stressed. Feels a lot pressure from his surroundings.”*

The CCE experts suggested that these patients depend greatly on predictable daily rhythms to regulate their behavior and emotions and to dare to engage in social interactions. The establishment of daily rhythms with sufficiently meaningful activities also often diminished patients’ substance abuse.

*Quote: “Team of sheltered housing facility is now supported by specialized mental health care and public mental health care.”*

#### Socially unacceptable behavior

The last refractory situation centered on a group of patients who came into conflict with society in a manner that their teams could not prevent. Patients in this situation were impulsive and naïve, with strong survival instincts. They regularly had problems with the police or judiciary. They felt that everything just seemed to happen to them and showed little insight into their problems, due partly to intellectual disability. They often had lacked sound social support systems from an early age. They ran the risk of being victims or perpetrators, depending on who they encountered in life. They were difficult for healthcare professionals to reach. Professionals, however, felt responsible for preventing incidents, yet being unable to do so they sometimes considered to discontinue treatment, which in turn increased the risk of incidents. At the start of consultation, professionals interpreted the challenging behavior from the perspective of a variety of psychological states, with diagnoses that varied over time. Most of these patients received (minimal) outpatient treatment, for which homelessness was a sustaining factor.

*Quote: “Patient lives in mental health care (MHC) sheltered housing facility. Is lonely, has little capacity and coping mechanisms. Often ends up in dangerous situations outside her living facility. Not well motivated for daytime activities. Many problems in primary support group. Addiction and abuse.”*

Problem explanations suggested by CCE experts emphasized the pattern of expulsion arising around these patients. They characterized this pattern as follows: the patients display annoying behavior to test whether other people may be trusted, which made the professionals turn away from them, confirming the patients’ idea that people cannot be trusted. Thus, the professionals inadvertently reinforced patients’ mistrust. In addition to attachment problems, patients often had mild intellectual disability or low social-emotional development, which contributed greatly to their struggle to understand the world and themselves. These patients were easily overwhelmed by rapidly changing fierce emotions, leading to dangerous situations. They retained their autonomy, but had difficulty doing so and accepted solid environmental contexts only to a limited extent.

*Quote: “Long-term, often negative, traces have been drawn in her existence that cannot be easily erased. The relationships with close people have not remained unscathed either; rapprochement and rejection alternated.”*

Possible solutions suggested by CCE experts emphasized that the disruption of the expulsion pattern required the establishment of unconditional relationships with these patients and the creation of safe living, work, and leisure environments in which the patients could acquire emotion-regulation skills and executive functions in close proximity to trusted others. In other cases, the environment and trusted others compensated the patients’ lack of these skills to prevent incidents. In these cases, risk assessment and examination of the patients’ development of conscience could be necessary.

*Quote: “More complete problem explanation for team. An structuring action program offered to team. Goes well with the patient. Reduction of medication. Involvement of a peer support worker. More contact with children and grandchildren. Tries to lose weight. Stimulated to have her walks.*

## Discussion

Reasons for consultation request can be divided in four exemplary situations which explain refractory cases. We found that 1) the cause of refractory mental health–related situations lies in patient–healthcare professional interaction, in which misjudgment plays a major role; 2) autism spectrum disorder and disharmonic developmental profiles are major factors in these interactions because of the risk of overestimation and incomprehension; and 3) healthcare professionals can improve these situations by consistently applying an interpersonal behavioral style based on unconditionality and closeness.

The most important conclusion of this study is that many refractory situations involve interaction patterns that inadvertently perpetuate or even exacerbate them. Patients tend to cope in ways that they are used to, which makes professionals feel inadequate and powerless, and this pattern continues in a vicious circle. To break it, professionals should act counterintuitively, seeking closeness to instead of distance from their patients. An outsider's perspective is required to see one's own part in the perpetuation of such patterns; recognizing that the situation is not improving and seeking consultation are signs of good practice [5]. Teams, patients, and their next of kin are emotionally involved in such situations, which limits the capacity for reflection [6, 9, 10]. New perspectives on the meaning of these situations facilitate choices that diverge from the patterns in which all actors have become entangled [5, 6, 11–13].

Professionals’ misjudgments observed in the consultations derived from their thinking in terms of (chronic) symptoms consistent with disorders requiring treatment. However, more treatment had already proven to be ineffective in these cases. The creation of a stable environment in which care giving is attuned to the patient’s (permanent) limitations is essential for patients’ recovery from prolonged overestimation [14, 15]. Because refractory situations always involve multiple problems, single-focus solutions usually do not work and cross-domain solutions should be considered.

The consequences of disharmonious developmental profiles, attachment problems, and ASD played prominent roles in three of the four exemplary situations presented in this study. These factors are frequent causes of overestimation and incomprehension. With an intellectual disability or attachment disturbance, disharmony arises between cognitive and socio-emotional developmental levels [15, 16]. It is only successful to appeal to a patient’s autonomy and responsibility when these are aligned with the person’s skills and needs. Thus, professionals must take such disharmony into account and to adapt their interpersonal behavioral styles accordingly. Investment in education on these themes may prevent the development of refractory cases.

Professionals’ adoption of an interpersonal behavioral style with attention to daily routines and meaningful activities was an essential part of all possible solutions to the refractory situations in this study. This behavioral style involves unconditionality and closeness, which can be challenging for professionals because they are sometimes counterintuitive. Coaching on the interpersonal behavioral style is strongly recommended [17].

Of note, formal testing of the described solutions, e.g. by randomized controlled trials, is beyond the scope of the current study. We intend to conduct a follow-up research in which we qualitatively will examine the four exemplary situations in more detail. With this study based on both the advisory reports of CCE experts and focus groups with involved professionals, next to kin and CCE experts we aim to provide a more robust insight in the patient characteristics and the implementation process of the proposed solutions.

The organization and formalization of opportunities for mental healthcare consultation, as done with the CCE in the Netherlands, provides several advantages for different stakeholders. First, consultation supports professionals in dealing with refractory cases. Second, it provides patients and their next of kin with opportunities to obtain independent consultation in situations in which patients’ quality of life is jeopardized and no agreement on possible solutions has been reached. Finally, the CCE is often asked to mediate in exceptional situations in which refractory cases reach the media and the Department of Health becomes involved.

Recent research on assumptions regarding consultation is scarce [6]. Thirty years of experience with consultations suggest that (financial) independence, interdisciplinarity, and a systemic approach are key factors for success. Reflection on interaction patterns and all actors’ contributions to interactions often yields new insight. Furthermore, because intellectual disabilities have a major impact on many consultations, mental health professionals can profit by enlarging their knowledge of these disabilities.

## Study strengths and limitations

The use of data from the CCE's database in this study provided an exceptional opportunity for the investigation of factors underlying the inability to provide adequate care to people with serious mental disorders across the entire Dutch MHS spectrum. Concurrently, it is important to realize that for CCE this is a naturalistic representative sample of cases but it is not a representative sample for all individuals receiving MHC. Moreover, this study has a number of limitations. The results of qualitative analysis may not be reproduced in the exact same way, since they are generated in part through the researchers' interpretations. We overcame this issue by using a large sample and combining the qualitative analysis with the calculation of descriptive statistics. Thematic content analysis relies on the systematic coding of open text. The researchers’ subjective influence in this coding is overcome by quality safeguards, such as co-author review and the use of expert panels.

## Conclusions

With this study, we have revealed interaction patterns that inadvertently perpetuate or even exacerbate refractory cases in MHS in the Netherlands, with the identification of four exemplary situations. These negative interaction patterns can be dispelled by reflecting on misinterpretations and investing in the development of more proactive interpersonal behavioral styles. In refractory cases, an outsider’s perspective gained through consultation yields promising insight for patients, their next of kin, and healthcare professionals.

## Data Availability

The dataset generated and/or analyzed during the current study are not generally publicly available due to the General Data Protection Regulation (GDPR), they can, however, be made available upon reasonable request to the corresponding author.

## Abbreviations

CCE: Center of Consultation and Expertise
MHS: Mental Health Service
MHC: Mental Health Care
CTS: Client Tracking System
ASD: Autism Spectrum Disorder
SED: Social-Emotional Development
